# Prevalence and characteristics of metaraminol usage in a large intensive care patient cohort. A multicentre, retrospective, observational study

**DOI:** 10.1016/j.ccrj.2025.100112

**Published:** 2025-06-23

**Authors:** Tarren Zimsen, Lachlan Quick, Gentry White, Rahul Costa-Pinto, Stephen Whebell, Jason Meyer, James McCullough, Kiran Shekar, Kevin B. Laupland, Mahesh Ramanan, Sebastiaan Blank, Alexis Tabah, Stephen Luke, Peter Garrett, Antony G. Attokaran, Aashish Kumar, Kyle C. White, the Queensland Critical Care Research Network (QCCRN)

**Affiliations:** a Intensive Care Unit, Princess Alexandra Hospital, QLD, Australia; b Intensive Care Unit, Townsville University Hospital, QLD, Australia; c School of Mathematical Sciences, Faculty of Science, Queensland University of Technology, QLD, Australia; d Department of Critical Care, University of Melbourne, VIC, Australia; e Department of Intensive Care, Austin Hospital, VIC, Australia; f School of Medicine and Dentistry, Griffith University, QLD, Australia; g Intensive Care Unit, Gold Coast University Hospital, QLD, Australia; h Mayne Academy of Critical Care, Faculty of Medicine, University of Queensland, QLD, Australia; i Adult Intensive Care Services, The Prince Charles Hospital, QLD, Australia; j School of Clinical Medicine, Faculty of Health, Queensland University of Technology, QLD, Australia; k Intensive Care Services, Royal Brisbane and Women's Hospital, QLD, Australia; l Intensive Care Unit, Caboolture Hospital, QLD, Australia; m Intensive Care Unit, Cairns Hospital, QLD, Australia; n Intensive Care Unit, Redcliffe Hospital, QLD, Australia; o Intensive Care Services, Mackay Base Hospital, QLD, Australia; p College of Medicine and Dentistry, James Cook University, QLD, Australia; q Intensive Care Unit, Sunshine Coast University Hospital, QLD, Australia; r Intensive Care Unit, Rockhampton Hospital, QLD, Australia; s Intensive Care Unit, Logan Hospital, QLD, Australia; t Intensive Care Unit, Queen Elizabeth II Jubilee Hospital, QLD, Australia

**Keywords:** Vasopressors, Hypotension, Metaraminol, Intensive care unit

## Abstract

**Background:**

Noradrenaline is the most prescribed vasopressor in intensive care units (ICUs). Although there is limited supporting evidence, metaraminol is often used as an alternative agent in some regions. We aimed to describe current practice and elucidate the factors associated with metaraminol prescription in a large cohort of ICU patients.

**Method:**

A multicenter, retrospective cohort study of granular, routinely collected electronic medical record–based clinical data was performed in 12 ICUs in Queensland, Australia, between January 1, 2015, and December 31, 2021. Patients who received at least four consecutive hours of either metaraminol or noradrenaline in the first 24 h of their ICU stay were included.

**Results:**

In total, 17,432 patients received single-agent vasopressor therapy and 1,963 (11.3 %) patients were administered metaraminol. For the entire cohort, the median age was 61 (interquartile range, IQR: 47–71), and the median Charlson Comorbidity Index was 3 (IQR: 1–5). The patients who received metaraminol had less ischaemic heart disease (5.5 % vs 7.6 %; p < 0.001) and were more likely to have localised cancer (16 % vs 14 %; p < 0.004). The patients receiving metaraminol were less likely to be ventilated on admission (39 % vs 73 %; p < 0.001) and had lower median Acute Physiology and Chronic Health Evaluation III scores (51 vs 56; p < 0.001). The median duration of metaraminol was 10 h (IQR: 6–18) and two-thirds (65 %) did not convert to noradrenaline infusion. After adjustment for confounders, after-hours admission (odds ratio, OR: 1.55; 95 % confidence interval [CI]: 1.40–1.71; p < 0.001), treatment limitation orders (OR: 1.35; 95 % CI: 1.10–1.64; p < 0.004), and admission to a regional ICU (OR: 1.47; 95 % CI: 1.27–1.68; p < 0.001) were independently associated with metaraminol use.

**Conclusion:**

Metaraminol is a widely used vasoconstrictor in Queensland ICUs. Patients who receive metaraminol have specific characteristics but are overall less unwell than patients who receive noradrenaline. Most patients who receive metaraminol do not require an alternative vasoactive medication.

## 1 Introduction

Hypotension is a common reason for admission to the intensive care unit (ICU) and, when untreated, is associated with severe complications, such as irreversible end-organ damage and eventually death.^1^ The first-line vasoactive agent for hypotension in most ICUs is noradrenaline, with vasopressin being the most common second-line agent, an approach supported by the guidelines.[2], [3], [4]

An alternative to noradrenaline and vasopressin is metaraminol,^5^ a synthetic, direct, and indirect sympathomimetic agonist acting mainly on alpha-1 adrenoreceptors but also with some beta-1 adrenoreceptors’ activity.^6^ It has gained significant preference for use in the anaesthetic context as a quick-onset vasoconstrictor agent that can offset the vasodilatory action of neuraxial, intravenous, and inhaled anaesthetic agents and can be delivered through a peripheral route.^7^^,^^8^

The British National Formulary and the Australian Injectable Handbook recommend using a central venous catheter for noradrenaline infusions; however, there are no such restrictions on using metaraminol.^5^ Given this, using metaraminol in a hypotensive patient has the advantage of safety through a peripheral venous cannula, which saves time and cost for central venous line insertion. Furthermore, the short- and long-term consequences of a central venous line, such as arterial puncture, thrombosis, and central line-associated bloodstream infection, can be avoided.

Though metaraminol is commonly used in some geographical jurisdictions,^5^^,^^9^ the evidence for its use is limited.^10^ There are no randomised control study data comparing metaraminol with other vasopressor agents in the ICU context for distributive shock. The best evidence at present is retrospective studies that only highlight the duration of use, delivery route and maximum doses.^9^^,^^11^ Interestingly, animal studies comparing metaraminol and norepinephrine in septic shock have highlighted no difference in many physiological parameters.^12^ Furthermore, physiological properties, such as increased pulmonary vascular resistance^13^ and tachyphylaxis^14^ with prolonged use, may impact metaraminol adoption.

To address the lack of clinical evidence for metaraminol, we aimed to describe current practice and elucidate the factors associated with its prescription in a large cohort of ICU patients.

## 2 Materials and methods

### 2.1 Study design

A multicenter, retrospective cohort design using granular, routinely collected electronic medical record–based clinical data was performed.

### 2.2 Study population

The study was conducted in 12 closed-model, mixed (medical and surgical) ICUs in Queensland, Australia, including five tertiary, three outer metropolitan, and four regional ICUs. It included all adult patients, age 18 years or greater, admitted between January 1, 2015, and December 31, 2021, and excluded all individuals admitted with palliative intent or transferred from another participating ICU. Inclusion in the study was limited to patients administered at least four continuous hours of either metaraminol or noradrenaline within the first 24 h of their ICU stay. Those treated with other vasoactive agents, either alone or combined with metaraminol or noradrenaline, were not considered for this analysis.

### 2.3 Data sources

Data were collected from all centres using the eCritical MetaVision™ (iMDsoft, Boston, MA, USA) clinical information systems and the Australian and New Zealand Intensive Care Society (ANZICS) CORE Adult Patient Database.^15^ The dataset included patient demographics, diagnoses, severity of illness, outcomes, hourly haemodynamic measurements, and vasopressor dosage. Primary and secondary diagnoses were obtained from the International Classification of Diseases 10 Australia Modification (ICD-10-AM) codes. At the same time, mortality data were collected from the Queensland Hospital Admitted Patient Data Collection and the Queensland Births, Deaths, and Marriage Registry. Admission diagnoses were categorised to optimise data accuracy and interpretability (Supplementary Methods, Table S1).

### 2.4 Definitions

The Charlson-defined comorbidities and index were calculated from the ICD-10 codes (Supplementary Methods, Table S2).^16^^,^^17^ The vasopressor dosage was converted to the norepinephrine equivalent, and the vasoactive inotropic score was calculated per protocol (Supplementary Methods, Tables S3–S4).^18^ After-hours commencement was determined if the first vasopressor was administered before 8 a.m. or after 8 p.m. The level of ICU was defined by the ANZICS adult patient database. Tertiary hospital is defined as a large tertiary teaching hospital. Metropolitan hospital is defined as a non-tertiary hospital in a large city or town. Rural or Regional hospital is defined as a small non-teaching hospital located outside large cities or towns.

### 2.5 Outcomes

The primary outcome is the prevalence of metaraminol infusion during the first 24 h of ICU admissions. The secondary outcomes were patient characteristics, time of metaraminol infusion, dose characteristics, and duration of infusion, the conversion rate from metaraminol to alternative vasoactive medication, length of ICU stay, ICU mortality, hospital mortality, and 1-year survival. The exploratory outcomes were factors associated with metaraminol use compared with patients who received noradrenaline.

### 2.6 Statistical analysis

Descriptive statistics were expressed as frequencies and proportions for categorical variables and medians with interquartile ranges (IQRs) for continuous variables with skewed distributions. Fisher’s exact and Pearson’s Chi-squared tests were used to compare the categorical variables. The Wilcoxon rank-sum test was used to compare continuous variables. Mixed-effect logistic regression models, including hospitals as a random effect, were developed to examine factors associated with metaraminol usage and conversion to noradrenaline. The variables used for analysis were determined a priori and reflected the clinical utility of available data. The results of the multivariable analysis were reported as odds ratios (ORs) with 95 % confidence intervals (95 % CIs). Given the large data set and multiple comparisons, a two-sided p value of <0.01 was chosen to indicate statistical significance.^19^ Statistical analyses were performed using R v.4.4.1.

### 2.7 Ethical considerations

The Metro South Hospital and Health Service Human Research Ethics Committee (HREC/2022/QMS/82024) approved this study, and an individual waiver of consent was granted.

## 3 Results

### 3.1 Baseline characteristics

During the seven-year study period, there were 89,117 adult admissions. Of these, 66,638 (74.8 %) were assessed for vasopressor use after excluding patients transferred from another hospital (19,670; 27 %) or admitted with palliative intent (543; 0.8 %).

**Fig. 1:**
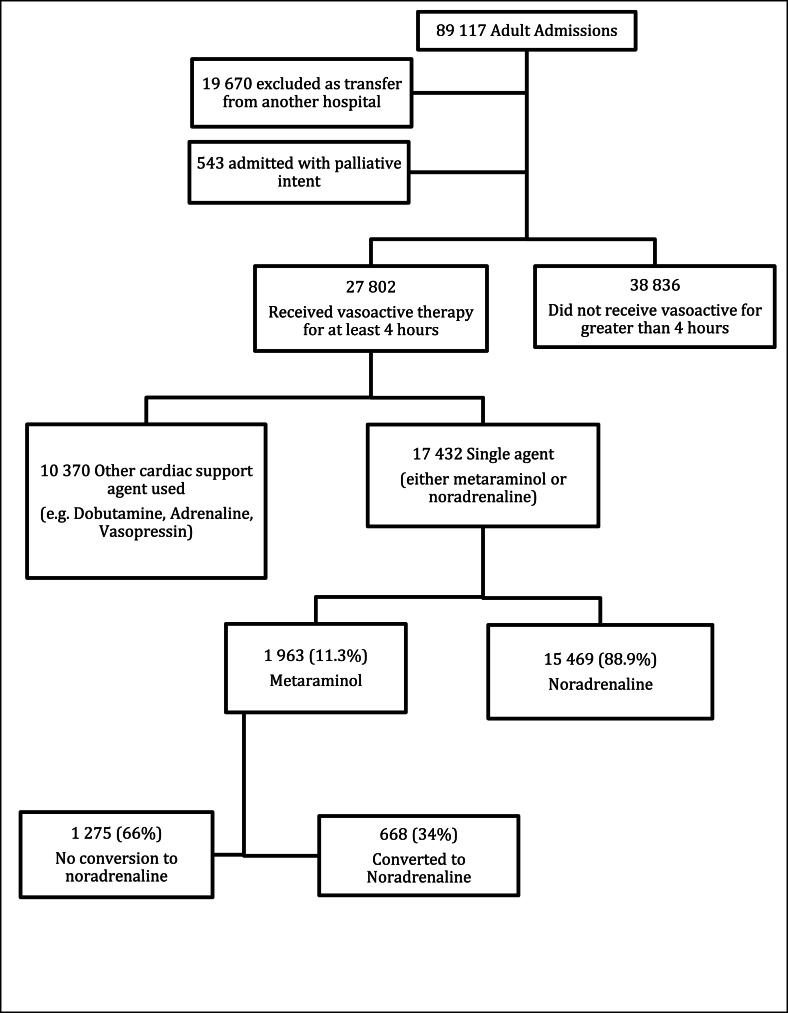
Flow chart describing patient selection.

In total, 27,802 patients (41.7 %) received vasoactive drugs during the first 24 h of their ICU admission. Among these patients, 17,432 (26.2 %) received either metaraminol or noradrenaline alone for at least four continuous hours. Of these, 1,963 patients (11.3 %) received a metaraminol infusion for a minimum of four continuous hours during the first 24 h of their ICU admission (Fig. 1).

### 3.2 Comparison of patients on metaraminol and noradrenaline

Of the 17,432 patients on a single vasoactive agent, the unadjusted results show the median age was 61 (IQR: 47–71) years, females were in the minority (6,831; 39 %), and the median Charlson Comorbidity Index was 3 (IQR: 1–5). Compared with patients who were on noradrenaline, those who received metaraminol were of the same age and had a similar body mass index and Charlson Comorbidity Index. However, they had less ischaemic heart disease (108; 5.5 % vs 1,177; 7.6 %; p < 0.001), less likely to have moderate to severe liver disease (30; 1.5 % vs 471; 3.0 %; p < 0.001), were more likely to have localised cancer (323; 16 % vs 2,167; 14 %; p < 0.004), and more likely to have cerebrovascular disease (213; 11 % vs 1,237; 8.0 %; p < 0.001). Patients receiving metaraminol were more likely to be admitted with neurological (290; 15 % vs 1,583; 10.0 %) or genitourinary diagnosis (119; 6.1 % vs 551; 3.6 %) and had lower Acute Physiology and Chronic Health Evaluation (APACHE) III score (51 vs 56; p < 0.001). (Table 1).

**Table 1:** Baseline characteristics.

Variable	Overall 17,432^a^	Metaraminol 1,963^a^	Noradrenaline 15,469^a^	p value^b^

Age (years)	61 (47, 71)	61 (47, 72)	61 (47, 71)	0.2
Female	6,831 (39 %)	834 (42 %)	5,997 (39 %)	0.001
Body Mass Index	27.7 (24.2, 31.6)	27.6 (23.9, 32.7)	27.7 (24.2, 31.6)	0.7
Charlson Comorbidity Index∗	3 (1, 5)	3 (1, 5)	3 (1, 5)	0.5
Co-morbidities				
Ischaemic heart disease	1,285 (7.4 %)	108 (5.5 %)	1,177 (7.6 %)	<0.001
Congestive heart failure	1,751 (10 %)	175 (8.9 %)	1,576 (10 %)	0.077
Chronic kidney disease	1,932 (11 %)	206 (10 %)	1,726 (11 %)	0.4
Peripheral vascular disease	952 (5.5 %)	90 (4.6 %)	862 (5.6 %)	0.07
Cerebrovascular disease	1,450 (8.3 %)	213 (11 %)	1,237 (8.0 %)	<0.001
Chronic pulmonary disease	1,784 (10 %)	212 (11 %)	1,572 (10 %)	0.4
Mild liver disease	622 (3.6 %)	45 (2.3 %)	577 (3.7 %)	0.001
Moderate to severe liver disease	501 (2.9 %)	30 (1.5 %)	471 (3.0 %)	<0.001
Diabetes	1,639 (9.4 %)	164 (8.4 %)	1,475 (9.5 %)	0.091
Diabetes with complications	2,483 (14 %)	241 (12 %)	2,242 (14 %)	0.008
Localised cancer	2,490 (14 %)	323 (16 %)	2,167 (14 %)	0.004
Metastatic cancer	1,041 (6.0 %)	117 (6.0 %)	924 (6.0 %)	>0.9
APACHE diagnosis group				<0.001
Cardiovascular	4,563 (26 %)	317 (16 %)	4,246 (27 %)	
Gastrointestinal	2,717 (16 %)	310 (16 %)	2,407 (16 %)	
Genitourinary	670 (3.8 %)	119 (6.1 %)	551 (3.6 %)	
Haematological	46 (0.3 %)	8 (0.4 %)	38 (0.2 %)	
Metabolic	1,331 (7.6 %)	176 (9.0 %)	1,155 (7.5 %)	
Neurological	1,873 (11 %)	290 (15 %)	1,583 (10 %)	
Other	529 (3.0 %)	88 (4.5 %)	441 (2.9 %)	
Respiratory	1,892 (11 %)	252 (13 %)	1,640 (11 %)	
Sepsis	2,139 (12 %)	225 (11 %)	1,914 (12 %)	
Trauma	1,672 (9.6 %)	178 (9.1 %)	1,494 (9.7 %)	
Admission circumstances				
Post elective surgery	5,855 (34 %)	651 (33 %)	5,204 (34 %)	0.7
Post rapid response	1,991 (12 %)	279 (14 %)	1,712 (11 %)	<0.001
Post cardiac arrest	935 (5.4 %)	62 (3.2 %)	873 (5.7 %)	<0.001
Severity of illness				
APACHE III score	55 (41, 73)	51 (38, 69)	56 (42, 74)	<0.001
Day of admission				
Any ventilation	12,036 (69 %)	772 (39 %)	11,264 (73 %)	<0.001
Maximum creatinine (μmol/L)	87 (66, 129)	83 (63, 122)	87 (66, 130)	<0.001
RRT	512 (2.9 %)	16 (0.8 %)	496 (3.2 %)	<0.001
Vasopressors	15,214 (87 %)	1,604 (82 %)	13,610 (88 %)	<0.001
NEE score (μg/kg/min)	0.04 (0.02, 0.08)	0.02 (0.01, 0.04)	0.04 (0.02, 0.08)	<0.001
Mean MAP (mmHg)	74.15 (70.08, 79.19)	73.38 (69, 78.80)	74.25 (70.23, 79.23)	<0.001
Maximum SOFA score	6 (5, 8)	4 (2, 6)	6 (5, 8)	<0.001
Maximum lactate (mmol/L)	2.1 (1.4, 3.5)	1.8 (1.2, 2.8)	2.1 (1.4, 3.6)	<0.001
ICU level				<0.001
Tertiary	12,477 (72 %)	1,256 (64 %)	11,221 (73 %)	
Outer metropolitan	1,842 (11 %)	125 (6.4 %)	1,717 (11 %)	
Regional	3,113 (18 %)	582 (30 %)	2,531 (16 %)	
Source of ICU admission				
Emergency department	5,603 (33 %)	603 (31 %)	5,000 (33 %)	<0.001
Operating theatre	8,916 (52 %)	999 (52 %)	7,917 (53 %)	<0.001
Ward	2,485 (15 %)	329 (17 %)	2,156 (14 %)	<0.001
Treatment goals on admission				0.030
Full active treatment	16,318 (94 %)	1,811 (92 %)	14,507 (94 %)	
Missing	3 (<0.1 %)	0 (0 %)	3 (<0.1 %)	
Treatment limitation order	1,111 (6.4 %)	152 (7.7 %)	959 (6.2 %)	

Abbreviations: APACHE = Acute Physiology and Chronic Health Evaluation; ICU = intensive care unit; MAP = mean arterial pressure; NEE = noradrenaline equivalent score; RRT = renal replacement therapy; SOFA = Sequential Organ Failure Assessment.

ICU Level - Tertiary hospital is defined as a large tertiary teaching hospital. Metropolitan hospital is defined as a non-tertiary hospital in a large city or town. Rural or regional hospital is defined as a small non-teaching hospital located outside large cities or towns.

a Median (IQR) or frequency (%).

b Wilcoxon rank-sum test; Pearson’s Chi-squared test; Fisher’s exact test.

On the day of admission, patients receiving metaraminol were significantly less likely to require ventilation (772; 39 % vs 11,264; 73 %; p < 0.001), had a lower Sequential Organ Failure Assessment score (4 vs 6; p < 0.001), and had lower maximum serum lactate (1.8 mmol/L vs 2.1 mmol/L; p < 0.001). Patients admitted to regional ICU were more likely to receive metaraminol than noradrenaline (582; 30 % vs 2,531; 16 %). Though statistically different, the source of ICU admission was comparable between the two groups, with operating theatre being the most common (Table 1).

### 3.3 Duration and dose of vasoactive medications

For the cohort that received metaraminol for any duration of time, the median time to commence treatment was 2 h (IQR: 1–5) and the median duration of vasopressor therapy was 17 h (IQR: 9–34). The median dose of metaraminol administered was 0.03 mg kg.h (IQR: 0.02–0.05) or 2.5 mg/h (IQR: 1.7–3.9), and the median maximum dose administered was 10 mg/h (IQR: 4–30). Most patients did not convert to noradrenaline infusion from metaraminol (1,275; 65.0 %). In this group, the median duration of metaraminol was 12 h (IQR: 7–20), and the median dose of metaraminol was 0.03 mg kg.h (IQR: 0.02–0.04). Of the patients who received metaraminol, 688 (35 %) converted to noradrenaline infusion. The median time to noradrenaline infusion from ICU admission was 10 h (IQR: 5–20), and the median dose of metaraminol in this group was 0.04 mg kg.h (IQR: 0.05–0.43). After the commencement of noradrenaline, the median time to cessation was 37 h (IQR: 22–57), and the median dose was 0.05 μg kg.min (IQR: 0.03–0.07). Of the patients started on metaraminol, 1,106 (56 %) were commenced after-hours (Table 2).

**Table 2:** Metaraminol usage.

Variable	Conversion to Noradrenaline			p value^b^
	Overall, N = 1,963^a^	No, N = 1,275^a^	Yes, N = 688^a^	

Afterhours commencement	1,106 (56 %)	761 (60 %)	345 (50 %)	<0.001
Time to metaraminol (hours)	2 (1, 5)	3 (1, 6)	2 (1, 5)	0.017
Time to noradrenaline (hours)	10 (5, 20)		10 (5, 20)	
Duration of either vasopressor (hours)	17 (9, 34)	12 (7, 20)	37 (22, 57)	<0.001
Duration of metaraminol (hours)	10 (6, 18)	12 (7, 20)	7 (5, 13)	<0.001
Duration of noradrenaline (hours)	0 (0, 15)		26 (14, 45)	
Dose of metaraminol, if any (mg/kg/h)	0.03 (0.02, 0.05)	0.03 (0.02, 0.04)	0.04 (0.02, 0.07)	<0.001
Dose of metaraminol, if any (mg/h)	2.5 (1.73, 3.92)	2.2 (1.59, 3.16)	3.36 (2.16, 5.45)	<0.001
Maximum dose of metaraminol (mg/h)	10 (4, 30)	4 (3, 7)	30 (6, 30)	<0.001
Dose of noradrenaline, if any (μg/kg/min)	0.05 (0.03, 0.07)		0.05 (0.03, 0.07)	
Time to conversion (hours)	6 (3, 14)		6 (3, 14)	

a Median (IQR) or frequency (%).

b Pearson’s Chi-squared test; Wilcoxon rank-sum test.

### 3.4 Outcomes

Compared with patients who received noradrenaline as the first vasopressor, an unadjusted analysis demonstrates that those who received metaraminol were less likely to require ventilation (44 % vs 77 %; p < 0.001) and less likely to require renal replacement therapy (3.6 % versus 8.1 %; p < 0.001) during their ICU admission. There was no statistically significant difference in mortality from 30 days to 1-year post-ICU admission. However, the unadjusted in-hospital mortality was lower in the metaraminol patients (8.0 % vs 9.7 %; p = 0.016). Patients who received metaraminol had a statistically significant shorter hospital stay (9 days versus 10 days; p < 0.001). There was no difference in hospital discharge destination between the two groups (Table 3).

**Table 3:** Outcomes following vasoconstrictor therapy.

Variable	Overall 17,432^a^	Metaraminol 1,963^a^	Noradrenaline 15,469^a^	p value^b^

Any ventilation	12,752 (73 %)	858 (44 %)	11,894 (77 %)	<0.001
Any RRT	1,322 (7.6 %)	70 (3.6 %)	1,252 (8.1 %)	<0.001
Any ECMO	34 (0.3 %)	6 (0.4 %)	28 (0.3 %)	0.6
ICU length of stay (days)	3 (2, 5)	3 (2, 4)	3 (2, 5)	<0.001
Hospital length of stay (days)	10 (6, 19)	9 (5, 16)	11 (6, 19)	<0.001
Died in ICU	1,215 (7.0 %)	115 (5.9 %)	1,100 (7.1 %)	0.040
Died in hospital	1,665 (9.6 %)	158 (8.0 %)	1,507 (9.7 %)	0.016
30-day mortality	1,805 (10 %)	184 (9.4 %)	1,621 (10 %)	0.13
90-day mortality	2,218 (13 %)	232 (12 %)	1,986 (13 %)	0.2
1-year mortality	3,036 (17 %)	325 (17 %)	2,711 (18 %)	0.3
Days alive without ventilation at 30 days	28 (25, 29)	29 (27, 30)	28 (24, 29)	<0.001
Days alive without vasopressors at 30 days	28 (26, 28)	28 (27, 28)	28 (26, 28)	<0.001
Days alive and out of hospital at 30 days	19 (7, 24)	21 (11, 25)	19 (7, 24)	<0.001
Days alive and out of ICU at 30 days	27 (23, 28)	27 (24, 28)	27 (22, 28)	<0.001
Discharge destination				0.3
Died	1,214 (7.0 %)	116 (5.9 %)	1,098 (7.1 %)	
Survived ICU	15,795 (91 %)	1,794 (91 %)	14,001 (91 %)	
Transferred to another hospital	179 (1 %)	22 (1.1 %)	157 (1 %)	
Transferred to other ICU	242 (1.4 %)	31 (1.6 %)	211 (1.4 %)	

Abbreviations: ECMO = extracorporeal membrane oxygenation; ICU = intensive care unit; RRT = renal replacement therapy.

a Median (IQR) or frequency (%).

b Pearson’s Chi-squared test; Fisher’s exact test; Wilcoxon rank-sum test.

### 3.5 Factors associated with metaraminol usage

Multivariable logistic regression analysis demonstrated several variables independently associated with metaraminol use compared with noradrenaline use as an initial vasopressor after admission to the ICU. The administration of ventilation (OR: 0.25; 95 % CI: 0.22–0.27; p < 0.001) or renal replacement therapy (OR: 0.35; 95 % CI: 0.21–0.54; p < 0.001) was associated with a lower likelihood of metaraminol usage. Admission post–rapid response team review (OR: 1.55; 95 % CI: 1.21–2.00; p < 0.001) and admission from the ward (OR: 1.46; 95 % CI: 1.03–2.1; p = 0.036) were associated with a higher likelihood of metaraminol use. The two values are different given 1991 patients were admitted following rapid response team review, and 2485 were admitted from the ward. If a treatment limitation was in place on admission (OR 1.35; 95 % CI 1.10–1.64; p < 0.004) there was a higher likelihood of metaraminol usage. Patients admitted to regional ICUs were more likely to receive metaraminol (OR: 1.47; 95 % CI: 1.27–1.68; p < 0.001). (Table 4).

**Table 4:** Factors associated with metaraminol use.

Characteristic	OR^a^	95 % CI^b^	p value

Age	1	1.00, 1.01	0.11
Charlson Comorbidity Index	0.98	0.95, 1	0.030
Source of ICU admission			
Emergency department	–	–	
Operating theatre	1.16	0.99, 1.35	0.068
Ward	0.84	0.66, 1.08	0.2
Elective surgery	0.94	0.80, 1.11	0.5
Admission post–rapid response	1.55	1.21, 2	<0.001
Admission post cardiac arrest	0.93	0.68, 1.24	0.6
APACHE III score	1	0.99, 1	0.004
Ventilation, day 1	0.27	0.24, 0.3	<0.001
Renal replacement therapy, day 1	0.29	0.17, 0.47	<0.001
Serum lactate (mmol/L), day 1	0.89	0.86, 0.91	<0.001
ICU level			
Tertiary	–	–	
Outer metropolitan	0.51	0.40, 0.63	<0.001
Regional	1.47	1.27, 1.68	<0.001
Treatment limitation order	1.35	1.10, 1.64	0.004
Afterhours commencement	1.55	1.40, 1.71	<0.001
Elective surgery ∗ ICU level			
Elective surgery ∗ outer metropolitan	1.76	0.91, 3.2	0.078
Elective surgery ∗ regional	1.39	1.04, 1.86	0.024

Abbreviations: APACHE = Acute Physiology and Chronic Health Evaluation; ICU = intensive care unit.

a OR = odds ratio.

b CI = confidence interval.

Elective surgery was not statistically significantly associated with a lower likelihood of metaraminol use (OR: 0.94; 95 % CI: 0.80–1.11; p < 0.5); however, when ICU level was considered, those admitted to a regional (OR: 1.76; 95 % CI: 1.04–1.86; p < 0.024) or outer metropolitan ICUs (OR: 1.76; 95 % CI: 0.91–3.20; p < 0.078) ICU after elective surgery had a higher likelihood of metaraminol use (Table 4).

### 3.6 Factors associated with conversion to noradrenaline

When comparing the patients that transitioned onto a noradrenaline infusion (668; 34.0 %) to those that remained on a metaraminol infusion (1275; 66.0 %), the multivariable logistic regression analysis highlights that requiring renal replacement therapy (OR: 6.57, 95 % CI: 2.86–19.1; p < 0.001) and ventilation (OR: 3.41, 95 % CI: 2.93–3.97, p < 0.001) on day 1 were independently associated with a conversion to noradrenaline (Table 5).

**Table 5:** Factors associated with conversion to noradrenaline.

Characteristic	OR^a^	95 % CI^b^	p value

Age	1	0.99, 1	0.3
Charlson Comorbidity Index	1.02	0.99, 1.06	0.2
Source of ICU admission			
Emergency department	–	–	
Operating theatre	1	0.80, 1.26	>0.9
Ward	1.46	1.03, 2.1	0.036
Elective surgery	0.68	0.53, 0.87	0.002
Admission post-rapid response	0.96	0.67, 1.39	0.8
Admission post cardiac arrest	0.73	0.48, 1.12	0.14
APACHE III score	1	1.00, 1.01	0.058
Ventilation, day 1	3.41	2.93, 3.97	<0.001
Renal replacement therapy, day 1	6.57	2.86, 19.1	<0.001
Serum lactate (mmol/L), day 1	1.17	1.12, 1.22	<0.001
ICU level			
Tertiary	–	–	
Outer metropolitan	2.43	1.77, 3.39	<0.001
Regional	1.08	0.89, 1.32	0.4
Treatment limitation order	0.82	0.61, 1.1	0.2
Afterhours commencement	0.69	0.59, 0.8	<0.001
Elective surgery ∗ ICU level			
Elective surgery ∗ outer metropolitan	0.52	0.23, 1.26	0.14
Elective surgery ∗ regional	0.94	0.63, 1.41	0.8

Abbreviations: APACHE = Acute Physiology and Chronic Health Evaluation; ICU = intensive care unit

a OR = odds ratio.

b CI = confidence interval.

## 4 Discussion

### 4.1 Key findings

Our multicenter study of over 17,000 critically ill patients with hypotension requiring either metaraminol or noradrenaline had several key findings. Firstly, the use of metaraminol was not uncommon and was administered in over 10 % of included patients. Secondly, patients who received metaraminol as an initial vasopressor had a lower severity of illness and received less invasive therapies. Thirdly, metaraminol was more likely to be administered in regional ICUs but used less in tertiary ICUs. Fourth, metaraminol was commenced early in the ICU admission and administered for less than 24 h. Fifth, nearly two in three patients who commenced metaraminol did not transition to noradrenaline during their ICU admission. Sixth, unadjusted outcomes such as mortality and discharge destination were similar among patients who received metaraminol or noradrenaline. Seventh, conversion from metaraminol to noradrenaline was associated with ventilation or renal replacement therapy on day 1 of ICU admission. Lastly, after adjustments for confounders, several variables were independently associated with using metaraminol. These data indicate that the decision to initiate metaraminol is directed by patient characteristics, location, time of day, and expected duration of illness.

### 4.2 Relationship to previous studies

To our knowledge, no studies describing metaraminol usage or examining the factors associated with its prescription have been previously published.

The single-centre study by Sardaneh et al. examined 152 critically ill patients admitted to an Australian ICU with comparable findings to ours.^9^ They demonstrated a median duration of metaraminol infusion of 7 h (IQR: 3–19) and a mean infusion rate of 4.0 mg/h. In an unadjusted analysis, the authors demonstrated that patients who received metaraminol monotherapy, compared with those with combination vasopressors, had a lower APACHE III score (58 vs 68, p < 0.001) and shorter duration of vasopressor therapy (12 vs 39 h; p < 0.001). ICU mortality was similarly very low (6 %) in this study, suggesting a lower acuity population selected to receive metaraminol.

Conversion to noradrenaline was lower in our patient cohort compared with the Sardaneh study (35 % vs 47 %). Reasons for conversion to noradrenaline may reflect an arbitrary dose threshold, time threshold or individual clinician preference. However, in our study, the median metaraminol dose in those who converted to noradrenaline was not significantly higher (0.04 v 0.03 mg kg.h), and the time to conversion was relatively short (median 6 h).

In a survey of critical care units in the United Kingdom, Grauslyte demonstrated metaraminol in the critical care setting was utilised in two-thirds of units.^5^ In the Australian setting, metaraminol appears to be frequently used in the Emergency Department setting for septic shock management, with prevalence ranging between 6 % and 42 %.[20], [21], [22] The lower prevalence of metaraminol use in our study (one in ten patients) may reflect lower utilisation in the ICU setting or earlier conversion to noradrenaline within 4 h of ICU admission.

### 4.3 Study implications

The study demonstrates current metaraminol usage and the characteristics associated with its prescription. The study does not compare the efficacy of metaraminol and noradrenaline and does not state the superiority of one agent over another. The study demonstrates that regional ICUs are more likely to use metaraminol, and metaraminol is more likely to be started after hours. The underlying reasons for this are unknown; however, these units may be staffed by less numbers or less experienced medical officers after hours, so central line insertion and noradrenaline administration have more barriers. Of all patients who commenced on metaraminol, almost two-thirds did not convert to noradrenaline and were weaned off vasopressors at 12 h. This finding suggests that patient selection for first-line metaraminol usage was accurate and may have resulted in fewer central line insertions for noradrenaline administration. The widespread usage of metaraminol within the studied population and the lack of difference in unadjusted outcomes between the two groups highlight the need for future comparative effectiveness research with both retrospective and prospective studies, including an assessment on comparative cost-effectiveness.

### 4.4 Strengths and limitations

This study has several strengths. Firstly, the cohort was sampled from an extensive, comprehensive ICU patient database covering nearly all ICU patient admissions in Australia’s third most populous state covering tertiary, metropolitan, and regional ICUs. This population is representative of the general Australian population and is generalisable to other high-income countries with established public health care critical care services. Secondly, the data were electronically extracted from a clinical information system and entered by clinical staff caring for patients and, therefore, free of bias.

We acknowledge some limitations. First, because this is an observational study, no causal inferences can be drawn from its findings and the associations described are only hypothesis-generating. Second, we cannot truly account for decision-making regarding why the patient was administered metaraminol. Therefore, there is a risk of confounding the outcomes from a possible primary selection bias. The demonstration of lower severity scores in patients who received metaraminol may be attributed to a conscious or subconscious clinician bias. Third, information that may influence the vasoconstrictor dose or duration such as steroid use, amount of fluid delivered, and the presence of invasive lines are not captured by the study. Fourth, the study does not differentiate between central and peripheral metaraminol or noradrenaline delivery. Fifth, given data were only available from ICU admission there are no data available on the vasoconstrictor used prior to entering the ICU. Sixth, the study does not provide detailed information on the direct or associated costs.

## 5 Conclusion

In this large, multicenter, retrospective observational study, metaraminol was commonly used in patients with hypotension requiring vasopressor therapy. Patients who received metaraminol as an initial vasopressor had lower severity of illness, received less invasive therapies, and were more likely to be from regional ICUs when compared with patients who received noradrenaline. Lastly, almost two-thirds of patients who started on metaraminol did not require additional vasopressor support. To assess whether the use of metaraminol infusions is beneficial would require comparative effectiveness research, both retrospective and prospective with a randomised controlled trial. These further studies will help guide future clinicians on the most appropriate vasoconstrictor therapy.

## Statement of ethics

This study was approved by the Metro South Hospital and Health Service Human Research Ethics Committee (HREC/2022/QMS/82024) and an individual waiver of consent was granted.

## Author contributions

The study conception and design (TZ, KW, LQ); data acquisition (all authors); analysis (KW, GW); interpretation of data (all authors); article drafting (TZ, KW, LQ, GW); article revision for important intellectual content (all authors); final approval of the version submitted for publication (all authors); agreement to be accountable for all aspects of the work in ensuring that questions related to the accuracy or integrity of any part of the work are appropriately investigated and resolved (TZ, KW).

## Data availability statement

Data cannot be shared publicly due to institutional ethics, privacy, and confidentiality regulations. Data released for research under Sect. 280 of the Public Health Act 2005 requires an application to the Director-General of Queensland Health (PHA@health.qld.gov.au).

## Funding sources

This research received no specific grant from funding agencies in the public, commercial, or not-for-profit sectors.

## Declaration of competing interest

The authors declare that they have no known competing financial interests or personal relationships that could have appeared to influence the work reported in this paper.
