# Supplementary material for: Prevalence and characteristics of metaraminol usage in a large intensive care patient cohort. A multicentre, retrospective, observational study
Source: Crit Care Resusc. 2025 Jun 23;27(2):100112. doi: 10.1016/j.ccrj.2025.100112 (PMC12242419; doi:10.1016/j.ccrj.2025.100112)
Supplement: Multimedia component 1 [file mmc1.docx]

| *Table S1. Creation of Diagnostic Groups* | |  |
| --- | --- | --- |
| **Category** | **APACHE-III J Diagnostic Codes** |  |
| Cardiovascular | 101-111  1202-1213 |  |
| Respiratory | 201-213,  1301-1304 |  |
| Gastrointestinal | 301-313  1401-1413 |  |
| Neurological | 401-410,  1501-1506 |  |
| Sepsis | 501-504 |  |
| Trauma | 601- 605,  1601-1605 |  |
| Metabolic | 701-704,2201 |  |
| Haematological | 801,802,2101 |  |
| Genitourinary | 901-903,1701-1705,  1801-1803 |  |
| Musculoskeletal | 1101,1102 |  |
| Other | ‘other codes’ |  |
| APACHE = Acute Physiology and Chronic Health Evaluation | |  |
|  |  |  |

**Prevalence and characteristics of metaraminol usage in a large intensive care patient cohort, multicentre, retrospective, observational study**

**Online Supplementary Tables**

*Table S2. Calculation of Charlson Co-morbidity Index*

| **Category** | **ICD-10 Codes** | **Weight** |
| --- | --- | --- |
| Ischaemic Heart Disease | I21, I22, I252 | 1 |
| Congestive Heart Failure | I50 | 1 |
| Peripheral vascular disease | I71, I790, I739, R02, Z958, Z959 | 1 |
| Cerebral vascular disease | I60, I61, I62, I63, I65, I66, G450, G451, G452, G458, G459, G46, I64, G454, I670, I671, I672, I674, I675, I676, I677 I678, I679, I681, I682, I688, I69 | 1 |
| Dementia | F00, F01, F02, F051 | 1 |
| Chronic pulmonary disease | J40, J41, J42, J44, J43, J45, J46, J47, J67, J44, J60, J61, J62, J63, J66, J64, J65 | 1 |
| Connective tissue disease | M32, M34, M332, M053, M058, M059, M060, M063, M069, M050, M052, M051, M353 | 1 |
| Peptic ulcer disease | K25, K26, K27, K28 | 1 |
| Mild liver disease | K702, K703, K73, K717, K740, K742, K746, K743, K744, K745 | 1 |
| Moderate-severe liver disease | K729, K766, K767, K721 | 3 |
| Diabetes | E109, E119, E139, E149, E101, E111, E131, E141, E105, E115, E135, E145 | 1 |
| Diabetes with complications | E102, E112, E132, E142 E103, E113, E133, E143 E104, E114, E134, E144 | 2 |
| Paraplegia | G81 G041, G820, G821, G822 | 2 |
| Chronic kidney disease | N03, N052, N053, N054, N055, N056, N072, N073, N074, N01, N18, N19, N25 | 2 |
| Localised cancer | C0, C1, C2, C3, C40, C41, C43, C45, C46, C47, C48, C49, C5, C6, C70, C71, C72, C73, C74, C75, C76, C80, C81, C82, C83, C84, C85, C883, C887, C889, C900, C901, C91, C92, C93, C940, C941, C942, C943, C9451, C947, C95, C96 | 2 |
| Metastatic cancer | C77, C78, C79, C80 | 3 |
| HIV | B20, B21, B22, B23, B24 | 6 |

| Table S3. Calculation of Norepinephrine Equivalent   \| **Drug** \| **Dose** \| **Norepinephrine Equivalent** \| \| --- \| --- \| --- \| \| Epinephrine \| 0.1 µg/kg/min \| 0.1 µg/kg/min \| \| Norepinephrine \| 0.1 µg/kg/min \| 0.1 µg/kg/min \| \| Dopamine \| 15 µg/kg/min \| 0.1 µg/kg/min \| \| Phenylephrine \| 1.0 µg/kg/min \| 0.1 µg/kg/min \| \| Vasopressin \| 0.04 units/min \| 0.1 µg/kg/min \|   Table S4. Calculation of Vasoactive Inotrope Score (VIS)   \| **VIS** = dopamine (µg/kg/min) + dobutamine (µg/kg/min) + [100 * norepinephrine (µg/kg/min)] + [100 * phenylephrine (µg/kg/min)] + [10 * milrinone (µg/kg/min)] + [10000 * vasopressin (units/kg/min) \| \| --- \| |
| --- | --- | --- | --- | --- | --- | --- | --- | --- | --- | --- | --- | --- | --- | --- | --- | --- | --- | --- | --- |
